# Fast Adipogenesis Tracking System (FATS)—a robust, high-throughput, automation-ready adipogenesis quantification technique

**DOI:** 10.1186/s13287-019-1141-0

**Published:** 2019-01-22

**Authors:** Chengxiang Yuan, Smarajit Chakraborty, Krishna Kanth Chitta, Subha Subramanian, Tau En Lim, Weiping Han, K. N. Bhanu Prakash, Shigeki Sugii

**Affiliations:** 1 Laboratory of Metabolic Medicine, Singapore Bioimaging Consortium (SBIC), Agency for Science, Technology and Research (A*STAR), 11 Biopolis Way #02-02, Singapore, 138667 Singapore; 2 Signal and Image Processing Group, Singapore Bioimaging Consortium (SBIC), Agency for Science, Technology and Research (A*STAR), 11 Biopolis Way #02-02, Singapore, 138667 Singapore; 3 Duke-NUS Medical School, Singapore, Singapore

**Keywords:** Adipocytes/obesity, Lipid droplets, Microscopy/fluorescence, Molecular imaging, Nuclear receptors, Adipose-derived mesenchymal stromal cells (ASC/ADSC/A-MSC), Adipogenesis and browning assay, iPS and ES cell, Oil Red O/Nile Red, High-throughput imaging-based quantitation

## Abstract

Adipogenesis is essential in in vitro experimentation to assess differentiation capability of stem cells, and therefore, its accurate measurement is important. Quantitative analysis of adipogenic levels, however, is challenging and often susceptible to errors due to non-specific reading or manual estimation by observers. To this end, we developed a novel adipocyte quantification algorithm, named Fast Adipogenesis Tracking System (FATS), based on computer vision libraries. The FATS algorithm is versatile and capable of accurately detecting and quantifying percentage of cells undergoing adipogenic and browning differentiation even under difficult conditions such as the presence of large cell clumps or high cell densities. The algorithm was tested on various cell lines including 3T3-L1 cells, adipose-derived mesenchymal stem cells (ASCs), and induced pluripotent stem cell (iPSC)-derived cells. The FATS algorithm is particularly useful for adipogenic measurement of embryoid bodies derived from pluripotent stem cells and was capable of accurately distinguishing adipogenic cells from false-positive stains. We then demonstrate the effectiveness of the FATS algorithm for screening of nuclear receptor ligands that affect adipogenesis in the high-throughput manner. Together, the FATS offer a universal and automated image-based method to quantify adipocyte differentiation of different cell lines in both standard and high-throughput workflows.

**Electronic supplementary material:**

The online version of this article (10.1186/s13287-019-1141-0) contains supplementary material, which is available to authorized users.

## Background

Fat, or adipose tissue, is well known as an important endocrine and energy storage organ. Abnormally high or low levels of the tissue cause predisposition to diseases such as diabetes, inflammation, and even cancer [1]. In vitro adipogenesis is an essential technique for the study of fat tissue and is frequently used in the study of human disease modelling and drug screening. The adipogenesis protocol is also an important tool for examining trilineage differentiation of mesenchymal stem cells (MSCs), which are actively investigated for clinical applications [2]. Adipose-derived stem cells (ASCs) are also popular clinical sources of MSCs, due to their ease of isolation and abundance [3, 4]. One of the standard criteria to determine authenticity of MSCs/ASCs is their good differentiation capacity into adipocytes, along with chondrocytes and osteoblasts. However, while the adipogenesis protocol is relatively well established, there is no systematic method established for robust quantification of cells undergoing differentiation.

Induced pluripotent stem cells (iPSCs) make up a recent powerful addition to the arsenal of the cell and lipid biologist. Due to their ability to de-differentiate from and also differentiate into cells from all three germ layers, they can be used to study adipogenesis in ways that were not previously possible. For example, iPSCs have been successfully used to study human lipodystrophy caused by the *BSCL2* gene mutation, which manifests as the near-complete absence of fat deposits in patients, resulting in the inability to extract adipose tissues from patients for analysis [5]. iPSCs can be used to overcome this issue by converting other cells, such as skin fibroblasts, into iPSCs, which can then be differentiated into adipocytes. This allows for the adipogenic effect of the mutations to be studied in vitro.

However, iPSCs also introduce new challenges in accurately quantifying adipogenesis. Despite the relatively high specificity of the available dyes in staining lipid droplets, they are susceptible to several kinds of off-target staining. This is especially visible when the dyes are applied to cell types that naturally form clumps such as embryoid bodies (EBs) derived from embryonic stem cells (ESCs) and iPSCs, due to nonspecific uptake of the dyes into the cell clumps. Additionally, ESCs/iPSCs also exhibit highly heterogeneous patterns of differentiation due to the random differentiation process of EB formation introducing additional uncertainty into the adipogenesis process [6]. Therefore, it is often difficult to quantify the fraction of ESC/iPSC-derived adipogenic cells in a precise and comprehensive manner.

Nile Red and Oil Red O are widely used neutral lipid stains which are useful in detecting the adipogenesis of various types of cultured cells [7]. While both dyes have been important in the analysis of adipogenesis in either cell culture or fixed tissue samples, there has been relatively little literature on accurate and reliable methods to quantify their signals in cell culture samples. Oil Red O is a member of the Sudan red family of dyes and has been used since the 1950s as an efficient stain of neutral lipids [8]. Its mechanism of action is based on the high solubility of Oil Red O in lipids versus the isopropanol solution in which the dye is dissolved [9]. Nile Red, also marketed as AdipoRed™, is a fluorescent dye identified in the 1980s. The dye fluoresces strongly while in lipophilic environments, but is quenched by aqueous environments, allowing it to act as a lipid-specific stain [7].

Existing protocols of quantification generally involve either dye extraction for Oil Red O [10] and fluorescence measurements for Nile Red stains by taking the ratio of nuclear stain strength to lipid stain strength and area [11, 12]. More often, however, the quantification is done by manual counting or visual estimation, which can be very time-consuming, prone to observer bias, and only capable of covering small samples sizes. This often leads to inaccurate measurements of adipogenesis due to the heterogeneous nature of differentiation requiring large samples to obtain statistically relevant results. The problem of these methods gets worse because standard adipogenesis protocols usually let cells become overconfluent, which makes identification of individual cells more difficult.

Currently available software techniques are capable of detecting and quantifying the round lipid droplets found in adipogenic cells [13, 14], but their application in ESCs/iPSCs are limited due to the high density of overlapping cells in EB outgrowths preventing the detection of lipid droplets. Therefore, these techniques are limited to detecting adipogenesis in monolayer cultures with relatively low confluence. Furthermore, these techniques utilise the absolute quantity of fluorescence or absorbance from a particular field and, as a result, are vulnerable to differences in cell density and changes in background fluorescence levels due to nonspecific staining.

To the best of our knowledge, this is the first image analysis technique which is specifically designed to tackle the difficult issue of measuring adipogenesis of ESCs/iPSCs by taking adipogenic degrees of individual cells into account, while maintaining its detection accuracy in monolayer cell lines such as ASCs and 3T3-L1 preadipocytes. Additionally, this technique can also recognise image data from different types of stains and be programmable to fully automated functions, which is useful for analysis of high-throughput drug screening.

## Materials and methods

### Cell culture and differentiation

All cells were cultured in various culture containers in humidified 5% CO_2_ incubators at 37 °C. Use of human patients-derived cells was conducted with informed consent obtained for each subject, approved by the National Healthcare Group Domain Specific Review Board, Singapore, and performed in accordance with its relevant regulations.

#### Differentiation of 3T3-L1 cells

Mouse 3T3-L1 preadipocytes were cultured following the conditions described by Yang et al. [15]. In brief, cells were proliferated to 70–80% confluence in T75 or T175 flasks using Dulbecco’s modified Eagle’s media (DMEM) supplemented with 10% heat inactivated newborn calf serum, and were passaged upon reaching 70% confluence using a 0.25% solution of trypsin/EDTA (Gibco). Differentiation was carried out on gelatin or poly-l-lysine-coated 96-well plates, by first seeding the cells (20,000 per well in 96-well plates) and letting them grow to confluence. On day 0, (2 days after the cells have reached confluence), adipogenesis was induced with 3T3 Induction Medium 1, consisting of DMEM supplemented with 10% heat-inactivated fetal bovine serum (FBS), 1 μM dexamethasone, 0.5 mM 3-isobutyl-1-methylxanthine (IBMX), and 1 μM insulin. On day 2, the cells had their media changed to 3T3 Induction Medium 2, consisting of DMEM, 10% FBS, and 1 μM insulin. On days 4 and 7, the cells had their media changed to 3T3 maintenance media (DMEM with 10% FBS), and the cells were ready for imaging on day 10.

#### Differentiation of human iPSCs and ESCs

Human iPSCs and ESCs were cultured following the conditions described by Sugii et al. [16]. Briefly, iPSCs and ESCs were maintained under feeder-free conditions in mTESR1 media (Stem Cell Technologies) on Matrigel (Corning) in 6-well plates. The cells had their media changed daily, except on weekends, when double the quantity of media was added and the cells were fed after 48 h. Passaging of the cells was performed every 5–7 days, usually when the iPSC clumps reached a size of 2 mm, and was carried out enzymatically by treatment of the cells with a 0.2% solution of dispase (Thermo Fisher) in DMEM-F12 medium for 6–7 min.

Differentiation of the iPSCs and ESCs was carried out following a modified version of the conditions described by Mohsen-Kanson et al. [17]. The cells were first developed into embryoid bodies (EBs) by detaching the cells using collagenase and transferring them onto ultra-low attachment 6-well plates. The EBs were then subjected to formation medium (EB medium) consisting of DMEM-F12 base media with 20% Knockout Serum Replacement (KOSR). On days 3, 4, and 5 following the detachment, the cells were fed with EB medium supplemented with 1 μM of retinoic acid. After the treatment, the cells had their media changed with EB medium every 2 days. On day 11, the EBs were plated onto gelatin-coated 24-well plates and maintained in EB media, changing the medium every 2 days. On day 20, the cells had their media changed to EB adipogenic differentiation media (DMEM/F12, 10% KOSR, 1 μg/ml insulin, 0.5 mM IBMX, 0.25 μM dexamethasone, 0.2 nM triiodothyronine (T3), and 1 μM rosiglitazone). The media were then changed every 3 days. On day 30, the cells were ready for imaging.

#### Derivation of mesenchymal stem cells from iPSCs and ESCs

The iPSC-derived MSCs were first treated in an identical manner to the EB protocol above, except that on day 20, the cells were dissociated completely with trypsin and transferred to T75 plates. The media was then changed to MSC maintenance medium (MSCmm), consisting of DMEM, 15% FBS, 0.5 ng/ml basic fibroblast growth factor (bFGF), and 1X Non-essential amino acids (Gibco). The cells were then passaged twice, with residual EB clumps removed by aspiration during media changes. The resulting iPSC/ESC-derived MSCs were then treated identically with ASCs below for culture purposes.

#### Immortalisation of human adipose-derived stem cells

Subcutaneous and visceral human ASCs (previously described in Ong et al. and Takeda et al. [18, 19]) were immortalised using the protocol detailed in Chen et al. [20]. Briefly, lentiviruses were produced using the pLVX-puro-Myc plasmid and the Lenti-X HT Packaging System. The culture media was collected after 48 h, filtered with a 0.2-μm filter, and then used to infect the ASCs with the presence of 4 μg/ml polybrene. The ASCs were then selected with 2 μg/ml puromycin for 3 days, following which they were continuously cultured in MSCmm. The immortalised ASCs retained normal differentiation levels up to passage 30 (data not shown).

#### Differentiation of human MSCs and ASCs

Human ASCs and iPSC-derived MSCs were proliferated to 80–90% confluence in T75 or T175 flasks using MSCmm and were passaged every 3–6 days upon reaching 90% conference by a 0.25% trypsin/EDTA solution.

Differentiation was carried out on gelatin or poly-l-lysine-coated 96-well plates, by first seeding the cells (20,000 per well in 96-well plates) and letting them grow to confluence. Two days after the cells have reached confluence (day 0), adipogenesis was induced with ASC induction media 1 (DMEM, 10% FBS, 1 μM dexamethasone, 0.5 mM IBMX, 1 μM insulin). On day 3, the cells were further induced with ASC induction media 2 (DMEM, 10% FBS, 1 μM insulin), and the cells then had their media changed on days 6 and 9 to maintenance media (DMEM, 10% FBS). To induce browning, differentiated ASCs on day 12 were treated with either 10 μM Forskolin or DMSO control for 6 h. The cells were ready for imaging on day 12.

### Drug screening using nuclear receptor ligand library

Differentiation was carried out as per the 3T3-L1 and iPSC-derived MSCs protocols above, except that after the cells have become confluent 2 days before adipogenic induction (day 2), the cells were treated with 10 μM each of nuclear receptor ligands (Enzo Life Sciences). The treatment was continued for a second time during the addition of the first differentiation medium till day 2, and the adipogenesis protocol was carried out as per normal to completion. Two independent experiments were carried out in 3T3-L1 cells, each in triplicate plates, and average adipogenic scores were calculated from total of six plates. Adipogenic scores from human iPSC-derived cells were estimated from a single plate.

### Fluorescent dye staining of cells

Cells were first washed with 1X phosphate-buffered saline (PBS) with Ca^2+^ and Mg^2+^, and then a mix containing 10 μg/ml of Hoechst 33342 and a 40× dilution of Nile Red (AdipoRed, Lonza) was used to stain the cells. The cells were covered in aluminium foil and incubated at room temperature on a rocking incubator for 15 min before fluorescence imaging. The phase contrast images were taken from the same fields and adjusted for the dynamic range (contrast adjusted in Photoshop to take up the entire dynamic range of the image).

### Oil Red O/haematoxylin staining of cells

Oil Red O stock solution was prepared by dissolving 30 mg of Oil Red O powder in 100 ml of 100% isopropanol and readied by dilution to 60% with water and passing through a 0.2-μm syringe filter.

Cells were washed with 1X PBS with Ca^2+^ and Mg^2+^ and then incubated with 10% neutral buffered formalin for 60 min. The cells were then washed with distilled water three times, incubated in 60% isopropanol for 5 min at room temperature, and then incubated in Oil Red O working solution for another 5 min. The cells were finally destained with distilled water until no more Oil Red O was dissolved into the water. Haematoxylin counterstain was applied by incubation with modified Harris haematoxylin for 1 min at room temperature, and then the cells were destained with distilled water until no more haematoxylin was dissolved into the water.

### Fluorescence measurements

Fluorescence intensity of adipocyte staining was measured with a Molecular Devices SpectraMax M2 plate reader for both 96 well and 24 well plates. For the Nile Red lipid stain, an excitation/emission wavelength of 485/572 nm was used, and for the Hoechst 33342 nucleus stain, an excitation/emission wavelength of 353/483 nm was used.

### High-throughput fluorescence imaging

Images were acquired using a Molecular Devices ImageXpress Micro system at × 10 or × 20 magnification. The DAPI fluorescence channel was used to image the nuclei stained by Hoechst 33342, while the FITC fluorescence channel was used to image the lipid droplets stained by Nile Red. The Nile Red fluorescence peak was also broad enough to be visualised using either the TRITC or the Texas Red fluorescence channels, with no significant losses in sensitivity.

### Colour imaging of Oil Red O stains

Colour images were acquired using a Nikon N-STORM microscope system at × 20 magnification. In order to convert the Oil Red O colour images into fluorescent images, the three colour channels of the image (red, green, and blue) were analysed in order to find regions of the image that were red (indicating presence of lipid droplets stained by Oil Red O) or blue (indicating presence of nuclei stained by haematoxylin). The difference images were then fed into the FATS algorithm to be analysed.

### FATS algorithm and image analysis

**Fig. 1:**
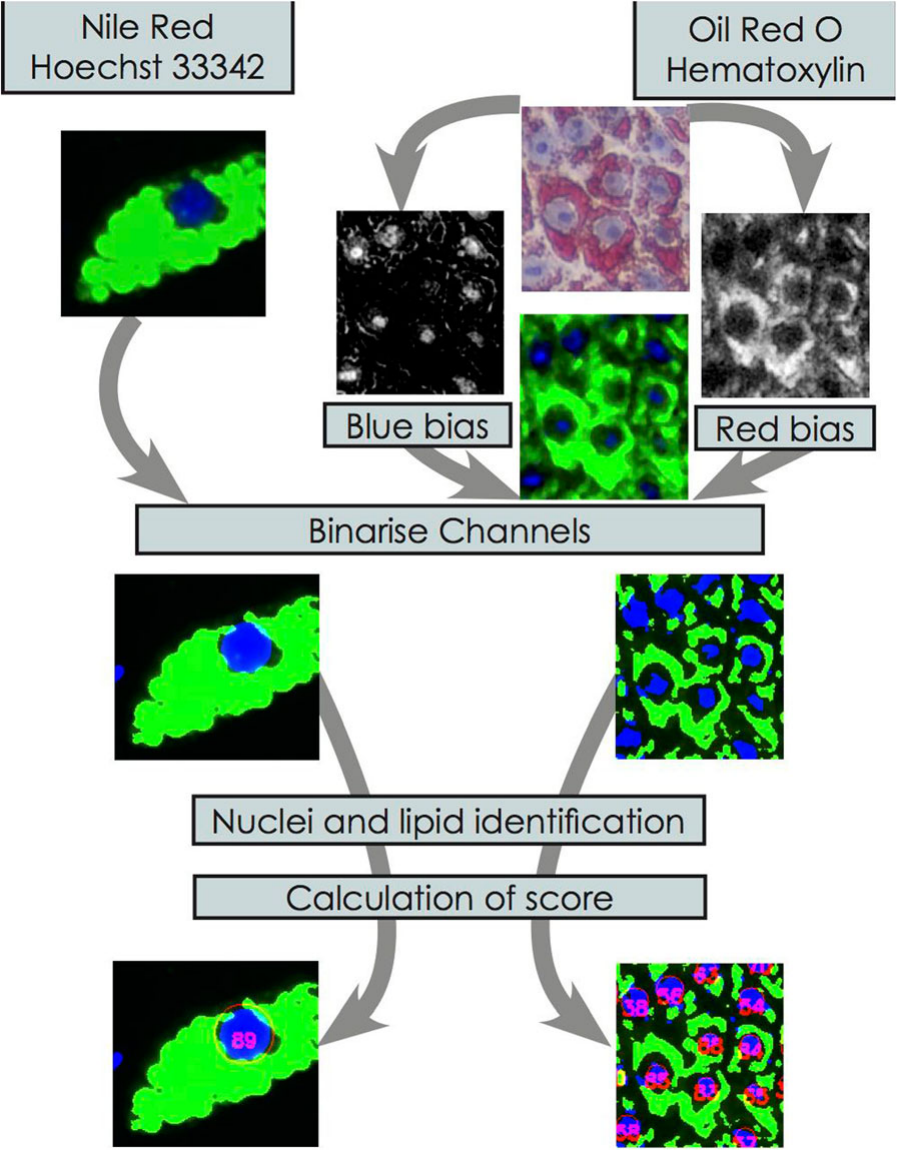
Flow chart of the FATS algorithm. The nuclei channel (blue) and the lipid channel (green) are generated from either the fluorescence or the colour images by calculation of the blue and red bias in the colour channels. The resulting two-channel image is then binarised, the nuclei identified, and the lipid scores calculated

The overall flow chart for the FATS algorithm and analysis is depicted in Fig. 1. We made use of lipid droplets staining by Nile Red and cell nuclei staining by Hoechst 33342 for our image-based algorithm, FATS. These fluorescent dyes would generally be much brighter than the background and have a sharp rise in brightness near the edges. However, when the cell densities become very high, which is often the case with overconfluency procedure of the adipogenesis protocol, the background staining also increases, in some cases getting brighter than the signal in other areas of the same image. To counteract this effect, a local intensity filter was used to detect local high-intensity regions, which greatly reduces the negative impact of background signals. The Python-OpenCV (https://github.com/itseez/opencv) and Mahotas [21] libraries were used to perform the image analysis. For ease of use, the iPython/Jupyter Notebook integrated development environment (IDE) [22] was used to simplify operation. The results were outputted and formatted using the Matplotlib libraries [23].

### Binarisation algorithm

Background subtraction and correction is an essential step in any automatic image analysis. A 2D Gaussian kernel G with dimensions *z* × *z* (where *z* is an odd integer) is generated from the image (Eq. 1). The value of *z* depends on the average sizes of the objects being detected, in this case the nuclei and groups of lipid droplets.

1 $$ {\mathrm{G}}_{x,y}=\alpha {e}^{-\frac{x^2+{y}^2}{2{\sigma}^2}},\sigma =0.3\left(\frac{z-1}{2}-1\right)+0.8 $$

The sizes of the kernels depend on the average sizes of the objects, and we found empirically that kernels with sizes two to three times the size of the average nucleus group produced the best results for 3T3-L1, MSC, and iPSC cells at high cell densities. In this case, we used a kernel size of 33 μm for nuclei and 66 μm for lipid droplet detection.

The factor α works as the correction factor that normalises sum of the entire Gaussian kernel to 1, where *e* is the base of the natural logarithm. The Gaussian kernel is convolved with overlapping sub-regions of the image In (Eq. 2), and the threshold value T_*x,y*_ for each sub-region is computed as the mean of the pixels in the convolution product.

2 $$ {\mathrm{T}}_{x,y}=\frac{1}{z^2}\sum \limits_{a=-\frac{z-1}{2}}^{\frac{z-1}{2}}\sum \limits_{b=-\frac{z-1}{2}}^{\frac{z-1}{2}}{\mathrm{G}}_{a,b}{\mathrm{In}}_{x-a,y-b} $$

This process is then iterated over the entire input image, producing the threshold image T.

3 $$ {\mathrm{Out}}_{x,y}=\left\{\begin{array}{c}255,{\mathrm{if}\ \mathrm{In}}_{x,y}>{\mathrm{T}}_{x,y}\\ {}0,\mathrm{otherwise}\end{array}\right. $$

Finally, the threshold image T is compared to the input image In (Eq. 3). If the threshold value for a given pixel is above that of the original input image, the pixel is considered to have a value of 255 (the maximum); otherwise, it has a value of 0. Since the thresholding takes into account the relative prominence of the pixel above its neighbours, it will be able to detect positive stains even in the presence of heavy background staining. While this step improves specificity of detection, it also loses some resolution due to the convolution process. As a result, the algorithm requires a pixel pitch of < 2 μm/pixel for optimal processing of the nuclei and lipid droplets.

After thresholding and removing the background, the nuclei were detected and segmented into individual regions. User-definable filters were applied on the aspect ratio (length to width) as well as sizes of the regions identified after binarisation. Regions that are too large, too small, or incorrectly shaped were excluded from the analysis. For example, dead cells often have highly misshapen nuclei distinguishable from live cells and could be removed through the filter. Then, a watershed algorithm was applied to divide nuclei that were joined together due to their proximity.

4 $$ \mathrm{S}={\mathrm{P}}_T\times {\mathrm{I}}_s $$

**Fig. 2:**
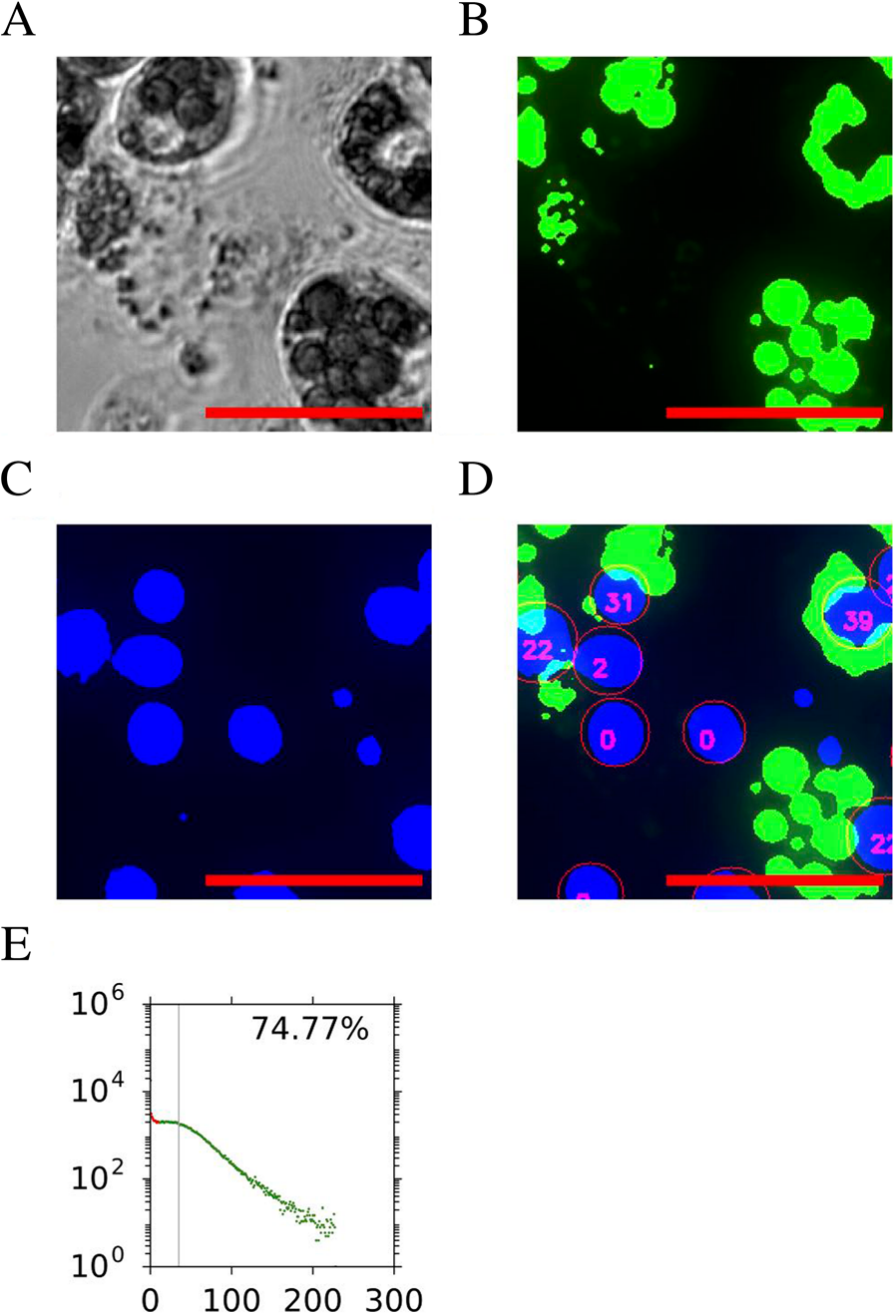
Representative analysis of 3T3-L1 cells after adipogenic stimulation. The representative phase-contrast image (**a**), binarised green lipid channel (**b**), and blue nuclei (**c**) channels are shown. The final calculated score for each of the detected nuclei is shown in **d**, where the number within each nucleus indicates the calculated lipid score for each of the nuclei. Note that blue-stained regions that were too small were excluded from the calculations. Finally, a histogram is created for all of the nuclei counted within the well, and the percentage of nuclei that have lipid scores above the threshold (in green dots) is indicated as adipogenic score in the upper right corner (**e**). For all the histograms, *X*-axis is the lipid score and *Y*-axis is the number of cells with the particular score. The vertical grey line indicates mean lipid fluorescence levels per nuclei for that cell population. Scale bars of images, 100 μm

The algorithm then searched for any lipid droplets in the vicinity of the nuclei using a user-definable distance outside of the bounding box of the nucleus. During our testing, we found that a radius of 3 μm was the optimal distance for balancing false positives and negatives when the cells were present in high densities. A lipid score of each nucleus S was calculated by a proportionate amount based on the percentage of area above threshold P_*T*_ and intensity of the stain I_*s*_ (Eq. 4). The lipid score of all the nuclei in the well was then tabulated, producing a histogram of adipogenic populations within the well. If the lipid score of a nucleus exceeds a pre-set gate threshold, the cell would be marked as differentiated, and otherwise the cell would be marked as non-differentiated. An example of analysis of differentiated cells is shown in Fig. 2, along with intermediate steps in the algorithm.

## Results

### FATS analysis of 3T3-L1 preadipocytes in the time-dependent manner

**Fig. 3:**
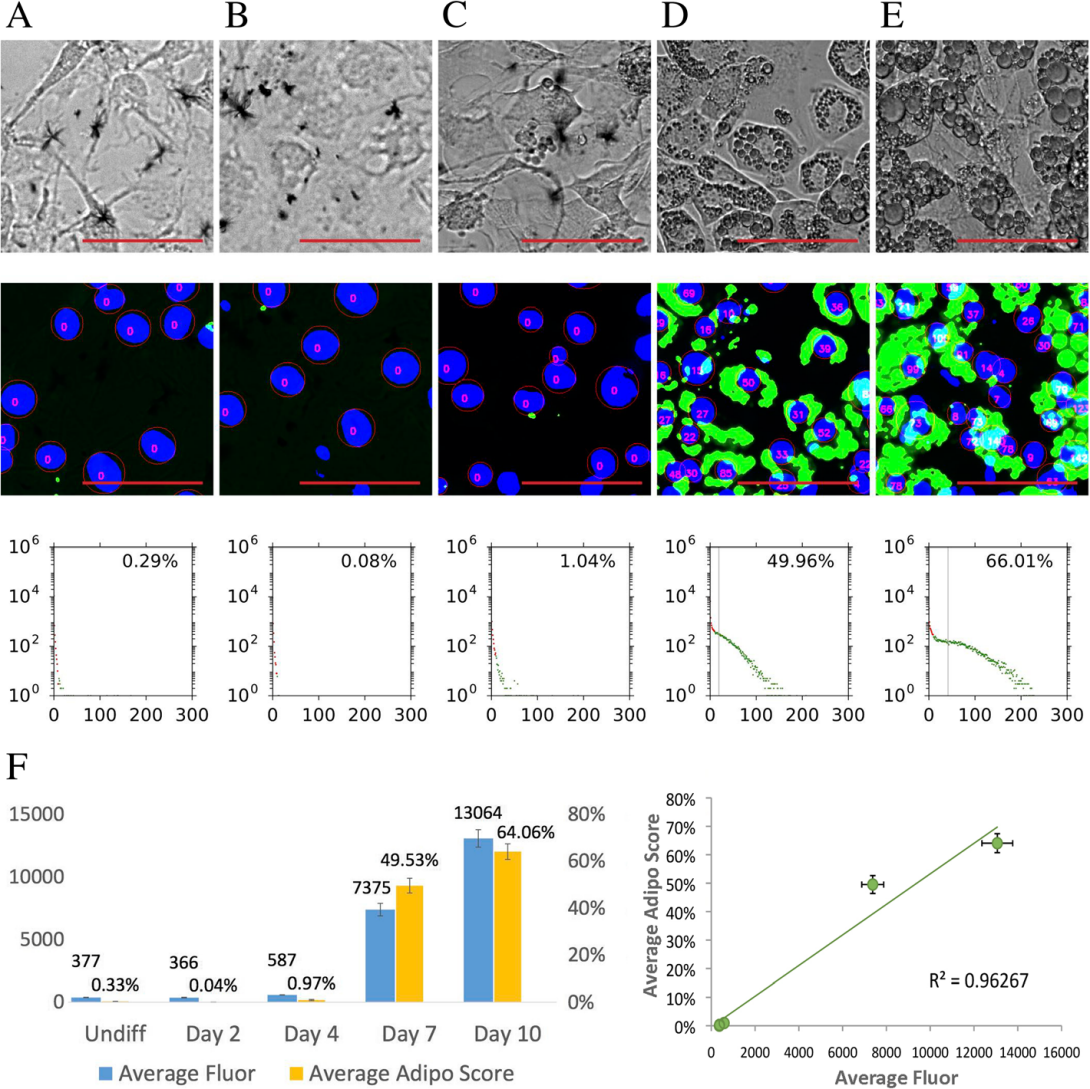
Time course assay of 3T3-L1 adipogenesis. The representative fields of phase-contrast (top row), nuclei and lipid droplets detection (middle row), and histograms from a whole well (bottom row) are shown for the five samples: undifferentiated (**a**), day 2 (**b**), day 4 (**c**), day 7 (**d**), and day 10 (**e**). Scale bars 100 μm. **f** The left graph shows average fluorescence measurements of Nile Red staining by spectroscopy (blue bars; left *y*-axis; *n* = 8) and average adipogenic scores (orange bars; right *y*-axis; *n* = 8–16) for each time points. The right graph indicates correlation between the spectroscopic fluorescence measurements (standard) and adipogenic scores (by FATS). Results are presented as mean ± standard error of mean (SEM)

We first tested the FATS algorithm on a time course assay using the 3T3-L1 preadipocyte line, which is commonly used to study adipogenesis. The cells were treated with the differentiation protocol for a varying number of days, then imaged and analysed (Fig. 3). The lipid score was computed for each nucleus by measuring the intensity and area of lipid droplets fluorescence around it. Histograms were created that indicate the lipid fluorescence level in *X*-axis and the count of cell nuclei in *Y*-axis. The adipogenic score was estimated by counting the percentage of nuclei having the lipid score above a gate threshold. The vertical grey line indicates mean lipid fluorescence levels per nuclei for that cell population.

There was a clear increase in the adipogenesis levels determined by the FATS algorithm during the time course. Consistent with known adipogenic development of 3T3-L1 cells, undifferentiated and day 2 cells showed minimal levels of lipid droplets (0.29% and 0.08%), a small number of day 4 cells started to develop lipid droplets (1.04%), substantial percentage of day 7 cells contained significant levels of lipid droplets (49.96%), and most of the day 10 cells have fully differentiated into mature adipocytes (66.01%). Additionally, the average adipogenic score of day 10 cells also increased versus day 7. In order to validate our method, fluorescence intensities of lipid droplet staining were measured by spectroscopy, which has been a commonly used standard method. The results indicate good correlation (*R*^2^ = 0.96267) of spectroscopic lipid droplet measurements with adipogenic scores by FATS analysis for all the time points (Fig. 3f). Besides the spectroscopic analysis, we have performed quantitative PCR to measure expression of five common adipogenic genes from days 0 to 12 of adipogenesis (*PPARG*, *FABP4*, *CD36*, *ADIPONECTIN* and *LEPTIN*; Additional file 1: Figure S1). Our correlation analysis indicates that there is also good association between each gene expression and adipogenic score (Additional file 1: Figure S1). It is also possible to modify the analysis method and measure mean or ranges of radius per lipid droplet. The distribution of lipid droplet radii indicates that adipogenesis is associated with increases in mean droplet sizes and emergence of larger droplets, especially between days 4 and 7, which is consistent with changes in the adipogenic scores (Additional file 1: Figure S2).

This result thus validates applications of the FATS in estimating percentage of cells having mature lipid droplets in the real time-dependent manner. Unlike the commonly practised means to manually and roughly estimate percentage of mature adipocytes by observers, the FATS analysis is capable of calculating the adipogenic percentage more in the subjective and precise manner.

### FATS can determine adipogenesis and browning levels of cell lines besides 3T3-L1

**Fig. 4:**
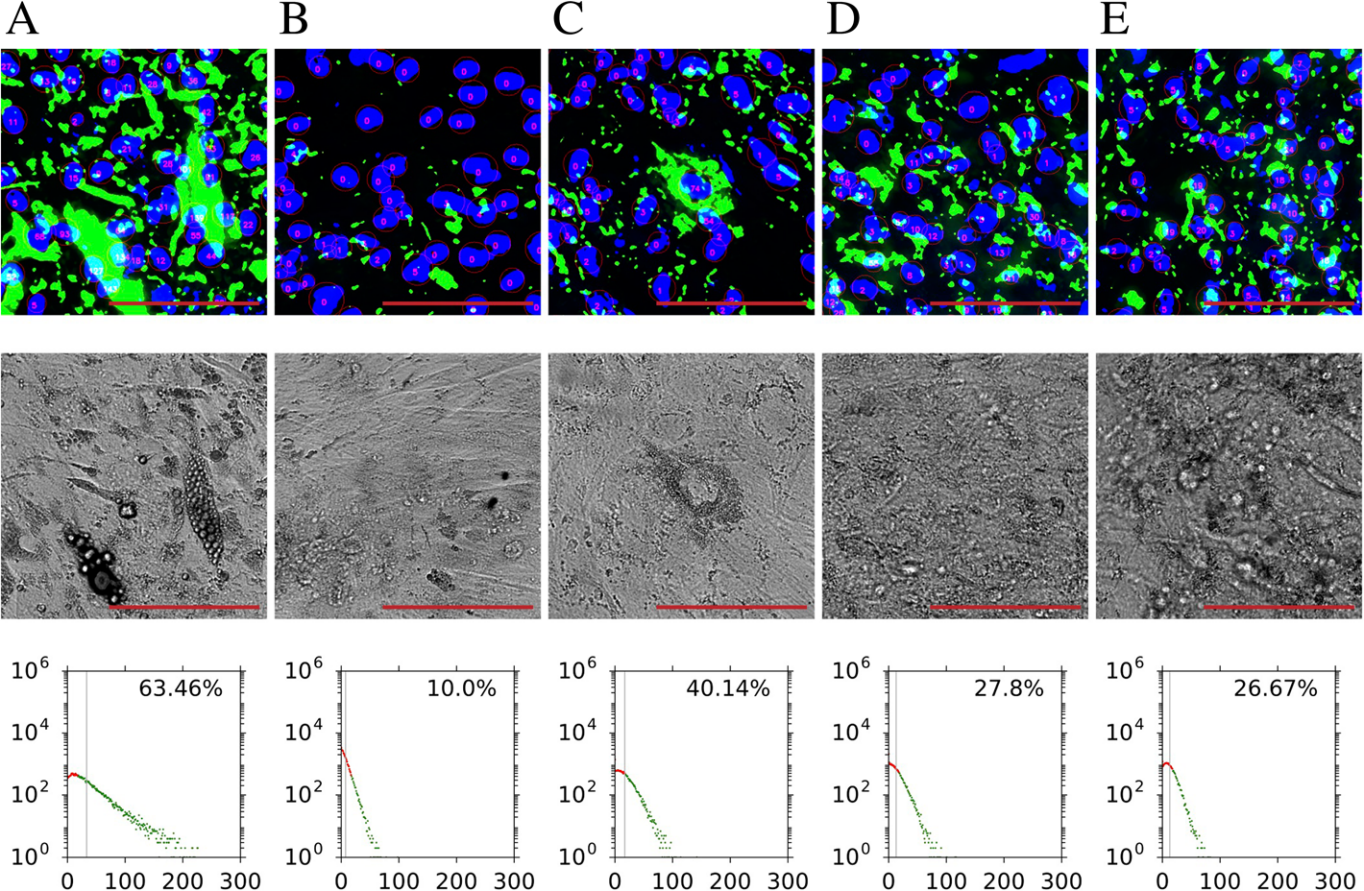
Adipogenesis analysis of human ASCs and iPSC-derived MSCs. The nuclei/lipid droplet detection of representative fields (top row), phase contrast fields (middle row), and whole well histograms (bottom row) are shown for the five samples: human immortalised subcutaneous ASCs (**a**), human immortalised visceral ASCs (**b**), human MSCs derived from iPSC line 1 (**c**), and human MSCs derived from iPSC line 2 (**d**), and human MSCs derived from HUES6 ESC line (**e**). Scale bars, 100 μm

In order to confirm applicability of the FATS algorithm in other cell lines than 3T3-L1, we subsequently ran the analysis on two human immortalised ASC cell lines and three MSC cell lines derived from iPSCs and ESCs (Fig. 4). It is known that under standard differentiation protocols in vitro, human subcutaneous fat-derived ASCs differentiate into adipocytes much better than visceral fat-derived ASCs [18, 19]. The FATS analysis proved and quantified these differences in subcutaneous ASCs (63.46%; Fig. 4a) and visceral ASCs (10.0%; Fig. 4b). We also tested and validated the FATS on adipogenesis of three MSC-like cell lines derived from pluripotent stem cell lines including iPSCs and ESCs (26.67–40.14%; Fig. 4c–e). The Nile Red staining used for the FATS is clearly more sensitive in detecting lipid droplets, compared to recognising lipid droplet structures in phase contrast images. Similar to 3T3-L1 cells, time course of adipocyte differentiation was studied in human iPSC-derived MSCs. The result indicates time-dependent increases in adipogenic scores during adipogenesis of these cells, consistent with those from 3T3-L1 cells (Additional file 1: Figure S3). In order to test if the FATS can be used to measure browning differentiation as well, subcutaneous-derived ASCs were differentiated into white adipocytes (Normal), followed by browning treatment (Browning). Additional file 1: Figure S4A shows the FATS analysis of representative staining images from normal and browning adipocytes. The results indicate that the FATS reliably quantifies significant drop in percentage of cells containing positive lipid droplets in browning adipocytes compared with white adipocytes, which is in line with standard quantification by spectroscopy (Additional file 1: Figure S4B and S4C). Together, these data provide applicability of the FATS algorithm beyond 3T3-L1 cell lines or standard adipogenesis.

### FATS is particularly useful for characterising adipogenesis of pluripotent stem cell lines

**Fig. 5:**
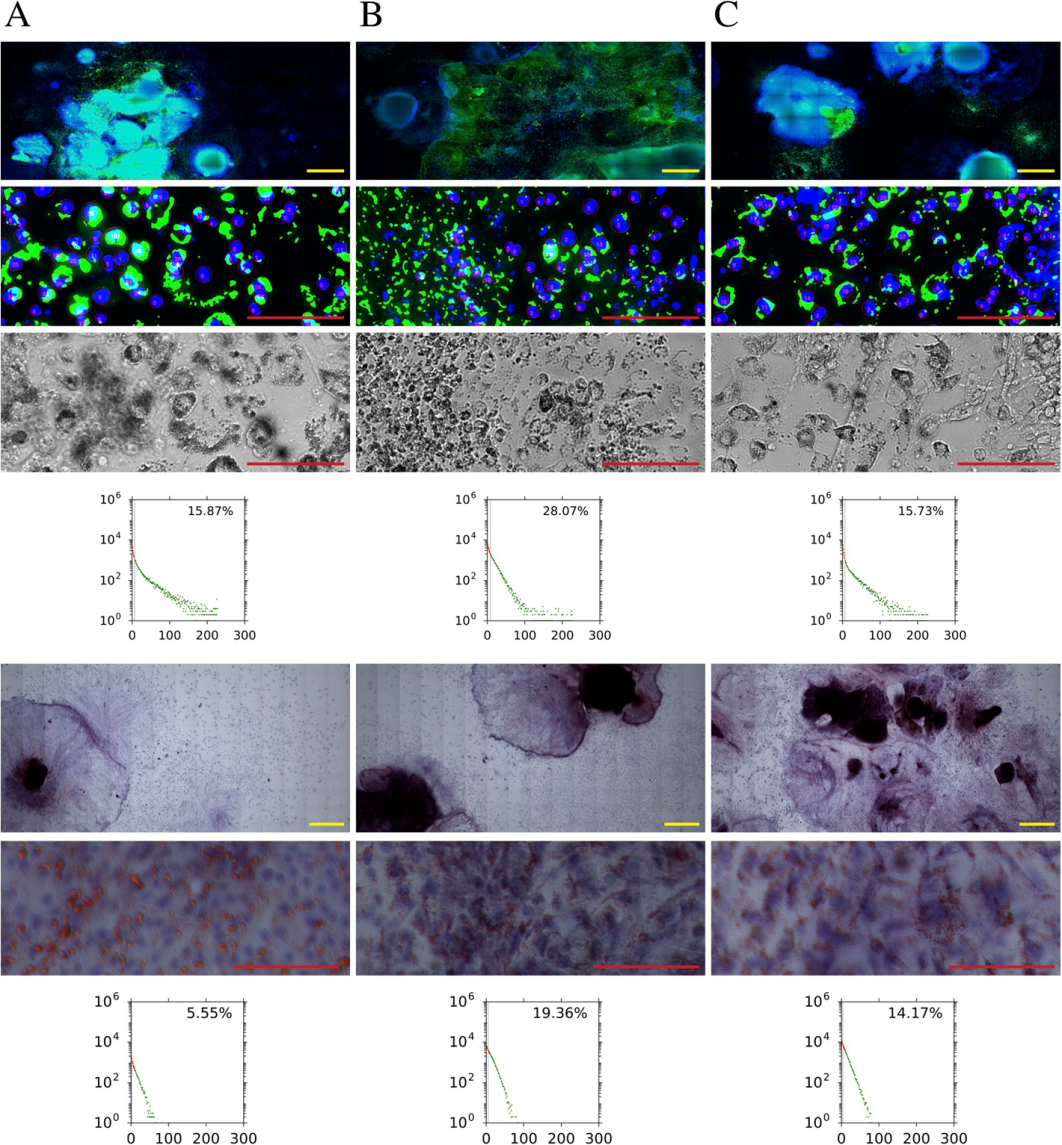
Analysis of direct adipogenesis of EBs derived from pluripotent stem cells. The fluorescence image analysis (top half) and Oil Red O image analysis (bottom half) of the entire well (top row) and of the representative microscopic field (middle rows) are shown along with their histograms (bottom row) for the 3 samples: human iPSC line 1 (**a**), human iPSC line 2 (**b**), human HUES6 ESC line (**c**). Phase contrast images are also shown as references for fluorescent analysis in the top half. The clump of cells to the left in the top row image of (**b**) are dead or dying, and therefore their nuclei fail to register and are excluded from consideration by the FATS analysis. Red scale bars: 100 μm, yellow scale bars: 1 mm

Next, we carried out the FATS analysis in direct adipogenesis of ESC/iPSC-derived EBs, which has been especially challenging to quantify. The result with Hoechst and Nile Red staining indicated successful measurement of ESC/iPSC-derived cells undergoing adipogenesis (15.73–28.07%; Fig. 5). As an additional benefit in the use of the FATS algorithm, dead cells are excluded for quantification by the analysis (see Fig. 5b as an example). Dead or dying cells often have highly misshapen nuclei and are therefore distinguishable from live cells when a nuclear stain is applied. The “small nucleus” filter of the FATS algorithm can then remove dead cells from consideration.

It is also possible to analyse with the FATS algorithm cells stained with more commonly used Oil Red O (for lipid droplets) and haematoxylin (for nuclei), albeit with significantly decreased sensitivity and inability to detect adipogenesis in high-density clumps of cells (Fig. 5). Comparing the two modes of imaging, it was observed that the fluorescence imaging was significantly superior to Oil Red O imaging as the fluorescence channels were independent of each other, whereas the red/blue bias were reduced if there was overlap between the red and blue channels in the colour image. This resulted in lower measured values for adipogenesis (5.55–19.36%) under Oil Red O images for all of the three samples.

**Fig. 6:**
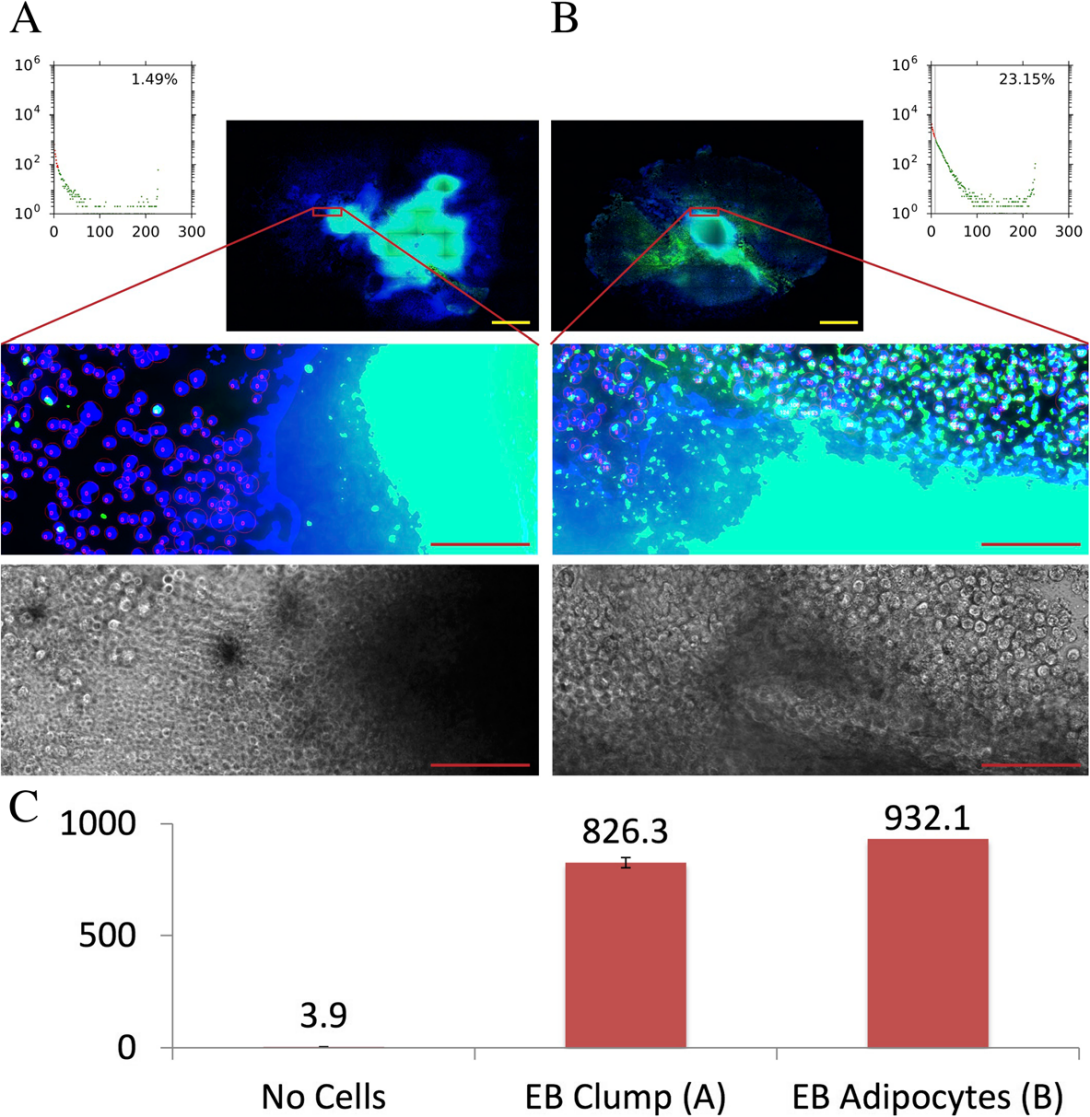
Improved accuracy of FATS analysis to estimate adipogenesis of EBs derived from pluripotent stem cells. An example of EB clump sample that is surrounded by non-differentiated fibroblastic cells spread out of EB and has little amount of differentiated adipocytes, yet adsorbs lipid droplets dye non-specifically (**a**). The FATS algorithm excludes such non-specific staining caused by cell clump, calculating an adipogenic score as 1.49%. The case of an EB sample that contains significant amount of differentiated adipocytes in the surrounding area gives rise to estimation of a good adipogenic score as 23.15% by FATS (**b**). This is in contrast to artificially high values of the EB clump sample (826.3) given by fluorescent spectroscopic reading (**c**). Red scale bars, 100 μm; yellow scale bars, 1 mm

In order to determine accuracy of the FATS analysis over the conventional quantification method, we investigated two sets of cell samples derived from human iPSCs. One sample set contains mostly non-differentiated fibroblastic cells that were spread out of a large EB clump in the centre (Fig. 6a). The second set contains well-differentiated adipocytes that were derived from and surrounding a smaller EB in the centre (Fig. 6b). The spectroscopic measurements of these two cell samples indicate high reading values of the EB clump sample relatively comparable to the EB adipocyte sample, due to non-specific adsorbing of Nile Red dyes by the EB clump structure (Fig. 6c). In contrast, the FATS analysis ignored non-specific staining caused by EB clump and more correctly calculated percentage of adipocytes for the EB clump sample (1.49%) and that for the EB adipocyte sample (23.15%). Together, these data support improved accuracy of the FATS system for estimation of adipogenesis derived from pluripotent stem cells.

### FATS is suitable for high-throughput image analysis of drug screening

**Fig. 7:**
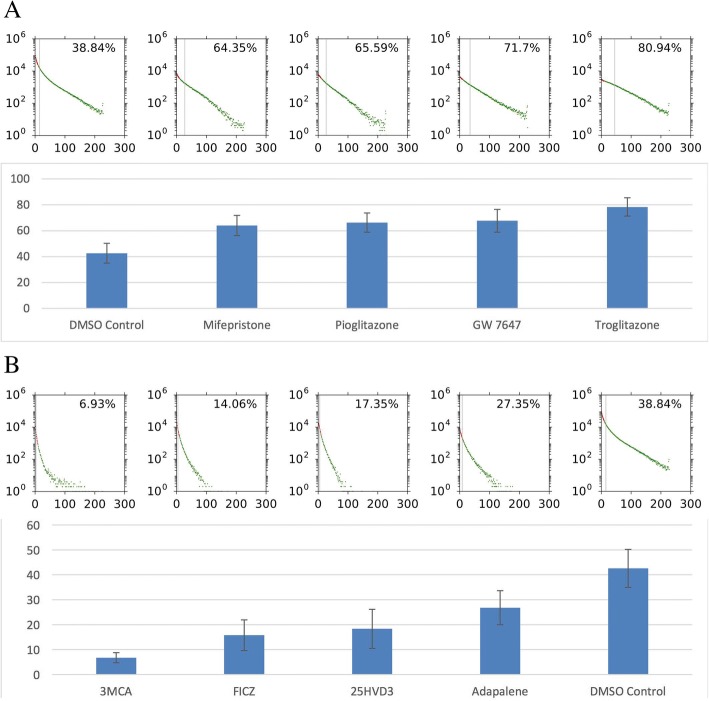
High-throughput screening assay of nuclear receptor ligands in 3T3-L1 cells analysed by FATS. Adipogenic scores for the top four samples (**a**) and the bottom four samples (**b**) are shown. Error bars are standard error of the mean (SEM) for *n* = 6 plates. Histograms indicate representative samples from a single plate

Finally, we did a proof-of-concept study to examine usefulness of the FATS in the high-throughput drug screening assay. As a test example, nuclear receptor ligand library composed of 76 compounds was chosen because a number of nuclear receptor superfamily members have been implicated in regulating the process of adipocyte differentiation [24–26]. The high-throughput assay was performed on 3T3-L1 cells, in two independent experiments, each consisting of triplicate plates (total of six plates). Although there was an inter-plate variation and a smaller amount of intra-plate variation, overall, the FATS algorithm was able to detect significant changes in the adipogenic percentage caused by drug treatments (Fig. 7, Additional file 1: Table S1). The top four hits included troglitazone (+ 34.99% on average compared to DMSO control), GW7647 (+ 27.80%), pioglitazone (+ 26.73%), and mifepristone (+ 24.16%) whereas the bottom four hits were 3-methylcholanthrene (3MCA; − 35.52%), 6-formylindolo [3,2-B] carbazole (FICZ; − 25.37%), 25-hydroxyvitamin D3 (25HVD3; − 22.21%), and adapalene (− 16.04%). In addition, library of nuclear receptor ligands were also investigated for adipogenesis of human iPSC-derived cells (Additional file 1: Figure S5 and Additional file 1: Table S2). The results indicate that all of the top four hits from the 3T3-L1 screening (i.e. troglitazone, GW7647, pioglitazone, and mifepristone) also increased adipogenesis whereas three out of four bottom hits (i.e. 3MCA, FICZ, and Adapalene, but not 25HVD3) decreased adipogenesis of iPSC-derived cells. This suggests that a good number of nuclear receptor ligands exhibit common adipogenic effects in different cell types. Collectively, these results demonstrate applicability of the FATS analysis to high-throughput image-based screening of adipogenesis.

## Discussion

While in vitro adipocyte differentiation has been widely used as an assay to study adipogenic processes and screen for potential therapeutic compounds, the methodology to quantify its differentiation degrees remains premature. There have been several methods previously proposed to analyse adipocyte biology. The most commonly used ones include gene expression, biochemical assays, microscopy, flow cytometry, and newer techniques such as laser-scanning cytometry [27] and coherent anti-Stokes Raman Scattering (CARS) [28]. Flow cytometry is still a widely used technique to analyse cells due to its highly quantitative nature, but the necessity of detaching cells from their growth surfaces prior to analysis makes flow cytometry difficult to apply to adipocytes [29]. In addition, adipocytes are very fragile and buoyant due to the presence of large low-density lipid droplets, and thus careful handling procedure and special setup are necessary, although an attempt has indeed been made to observe adipogenesis using flow cytometry [11].

The necessity of dissociating cells during one-dimensional flow cytometry techniques led to the development of laser-scanning cytometry (LSC), which has many similarities with fluorescence microscopy. However, fluorescence microscopy is still superior to LSC in that it can achieve much higher rates of measurement. For example, a single image at the recommended setting of × 20 magnification can cover 500–1000 cells per second, and the rate can be further increased if lower magnifications are used. In comparison, LSC achieves up to 5000 cells per minute under optimum conditions [30]. While the dynamic range of measurements is lower in fluorescence microscopy, the observed dynamic range is more than sufficient to detect well-stained adipocytes.

Dye-free detection from phase contrast/bright field images has also been proposed as a method which can detect lipid droplets in adipocytes by Varinli et al. [14] and by Sims et al. [31]. This method uses contour edge detection to identify the circular lipid droplets in monolayer culture cells such as 3T3-L1 cells and can quantitate the size and quantity distributions of lipid droplets within the image analysed. However, as shown in the corresponding phase contrast/bright field images, authentic lipid droplet structures are sometimes invisible and may thus not be recognised by the phase contrast-based analysis. Another main drawback of this method is that high cell densities and the presence of partially overlapping cells, due to ‘overconfluent cells’ nature of standard adipogenesis protocols, can confound the contour edge detection. This is especially problematic for analysis of ESC/iPSC-derived adipogenesis because in the vicinity of EB outgrowths, lipid droplet counting becomes very difficult, and fluorescence microscopy is much more effective at detecting adipogenesis in such cases. Another paper by Surdo et al. [32] used fluorescent dyes Hoechst and Nile Red for specifically analysing bone marrow-derived mesenchymal stem cells. It made use of the macro function of existing NIS-Elements software, where the Nile Red signals are enhanced to the level enough to overlay the nuclei signals. While the method is convenient to run and likely suitable for comparable analysis of similar samples, it can oversimplify the estimation, not reflecting actual changes in lipid droplet accumulation during adipogenesis and particularly problematic for estimating ES/iPS-derived adipogenesis. There was no evidence shown for correlation with conventional analytic methods. In contrast, our technology does not depend on particular commercial software (capable of taking existing images), not require lipid droplets overlaid with nuclei (instead taking their proximity to nuclei into account), and can deal with other staining methods such as Oil Red O and haematoxylin. The FATS analysis overcomes difficulties of measuring ESC/iPSC adipogenesis by disregarding the areas of cell clumps that can non-specifically adsorb lipid droplet dyes, as exemplified by Fig. 6. More technically, the system excludes the multilayers from consideration due to the inability to correctly resolve nuclei. This is because the multilayered clumps in the middle of EB differentiation take up dyes non-specifically, and therefore, they are usually stained a uniform cyan colour (maxed out blue and green fluorescence). This means that the post-segmentation nuclear regions and lipid regions will exceed the maximum pixel size limit and therefore become excluded from the calculations. As the outgrowth layers become less highly confluent (generally ≤ 3 layers) and the nuclei become resolvable, classification will then proceed as per normal.

The global obesity epidemic has resulted in the increasing need for drugs which can be used to treat obesity and associated complications. The identification of substances that increase or decrease the process of adipogenesis can offer further insight into the adipogenesis mechanism and therapeutic responses of adipose tissue. The effects of these drugs could manifest through either increase in cell size (hypertrophy) or numbers (hyperplasia). Both of these effects can be measured in a high-throughput manner in vitro using the FATS algorithm. As a proof of concept, we demonstrated usefulness of the FATS in assessing the effect of ligands for nuclear receptor superfamily using both 3T3-L1 preadipocytes and human iPSC-derived MSCs. Among the top hits, troglitazone and pioglitazone are well-known agonists for PPARγ, which is a master regulator of adipogenesis [33, 34]. Similarly, PPARα agonist GW7467 was previously shown to promote adipogenesis [35]. The effect of progesterone receptor antagonist mifepristone/RU486 is less clear, but this compound was reported to induce adiponectin production and glucose uptake in differentiated adipocytes [36]. In contrast to top four hits, the effects of three out of four bottom hits—aryl hydrocarbon receptor agonists, 3MCA and FICZ, and retinoid adapalene—in adipogenesis are unclear and warrant further study. In contrast, 25HVD3 was shown to inhibit adipogenesis and its level in circulation is inversely correlated with obesity in human [37, 38]. However, the inhibitory effect of 25HVD3 was not observed in human iPSC-derived cells. Together, these data validate the FATS analysis for identifying known and novel compounds that affect adipogenesis in the high-throughput manner.

Our analytical method can be modified to measure other parameters. In one modification, we demonstrated changes of size distribution and mean per lipid droplet during adipogenesis. Despite potential versatility, our method can be further improved to fully realise the potential. For example, measurement of mean fluorescence intensity per LD was relatively challenging due to heterogeneities in staining degrees, brightness, and contrast across different image fields even within a well. This is why, we made binarisation and background subtraction/correction of images, which resulted in normalisation of fluorescence intensities. Authentic estimation of other potential parameters such as LD counts per cell and total LD areas per cell would be possible if we find and incorporate a good cell surface staining dye that works well in mature adipocytes. Meanwhile, *X*-axis of our FATS histograms indicates arbitrary LD fluorescence levels surrounding each nucleus, which may be approximation to measures of LD area per cell. We believe that future improvement depends on better image acquisition system from imaging equipment and reliable cell surface demarcation. This is particularly important for estimating ESC/iPSC-derived adipogenesis where non-specific staining areas due to large cell clumps and high cell densities must be unequivocally excluded.

## Conclusions

In conclusion, we have demonstrated that the FATS algorithm is a robust approach for measuring adipogenesis in a wide range of cell types and that it can be universally used as a high-throughput image-based screening method for detecting drugs that affect the process of adipogenesis. The system is versatile, accurate, and capable of distinguishing dead cells, non-specific cell clump, and immature adipocytes with scant lipid droplets. Although we demonstrate in this paper that the FATS algorithm can analyse images of cells stained by Nile Red or Oil Red O, it can be applicable to other lipid droplet staining methods, such as those by lipophilic dyes of BODIPY or LipidTOX species. While the FATS can exclude dead cells based on the misshapen nuclei, it is also possible to modify the algorithm with a live/dead staining, such as propidium iodide on a third fluorescence channel, to completely rule out the likelihood of including dead cells.

## Additional file

Additional file 1: **Figure S1.** Correlation of adipogenic scores and adipogenic genes expression in 3T3-L1 cells. **Figure S2.** Sizes per lipid droplet from Nile Red staining were estimated with the modified method using background subtraction and circular Hough transform during adipogenesis of 3T3-L1 cells. **Figure S3.** Adipogenesis time course of human iPSC-derived MSCs. **Figure S4.** FATS analysis of browning in subcutaneous fat-derived ASCs. **Figure S5.** High-throughput adipogenic screening assay of nuclear receptor ligands using human iPSC-derived MSCs was analyzed by FATS. **Table S1.** List of all the nuclear receptor ligands that were tested for high-throughput screening assay for adipogenesis in 3T3-L1 cells. **Table S2.** Results of high-throughput screening assay for adipogenesis of human iPS-derived MSCs in a single plate using nuclear receptor ligands library. **Table S3.** List of RT-qPCR primers-Oligos 5’ to 3’.(DOCX 5748 kb)

## Abbreviations

25HVD3: 25-Hydroxyvitamin D3
3MCA: 3-Methylcholanthrene
ASC: Adipose-derived stem cell
CARS: Coherent anti-Stokes Raman Scattering
EB: Embryoid body
ESC: Embryonic stem cell
FATS: Fast Adipogenesis Tracking System
FICZ: 6-Formylindolo [3,2-B] carbazole
iPSC: Induced Pluripotent stem cell
LSC: Laser-scanning cytometry
MSC: Mesenchymal stem cell
