# Supplementary material for: Fast Adipogenesis Tracking System (FATS)—a robust, high-throughput, automation-ready adipogenesis quantification technique
Source: Stem Cell Res Ther. 2019 Jan 22;10:38. doi: 10.1186/s13287-019-1141-0 (PMC6341617; doi:10.1186/s13287-019-1141-0)
Supplement: Supplementary file 1 — Figure S1. Correlation of adipogenic scores and adipogenic genes expression in 3T3-L1 cells. Figure S2. Sizes per lipid droplet from Nile Red staining were estimated with the modified method using background subtraction and circular Hough transform during adipogenesis of 3T3-L1 cells. Figure S3. Adipogenesis time course of human iPSC-derived MSCs. Figure S4. FATS analysis of browning in subcutaneous fat-derived ASCs. Figure S5. High-throughput adipogenic screening assay of nuclear receptor ligands using human iPSC-derived MSCs was analyzed by FATS. Table S1. List of all the nuclear receptor ligands that were tested for high-throughput screening assay for adipogenesis in 3T3-L1 cells. Table S2. Results of high-throughput screening assay for adipogenesis of human iPS-derived MSCs in a single plate using nuclear receptor ligands library. Table S3. List of RT-qPCR primers-Oligos 5’ to 3’.(DOCX 5748 kb) [file 13287_2019_1141_MOESM1_ESM.docx]

**Additional file 1**

## Fast Adipogenesis Tracking System (FATS) – A robust, high throughput, automation-ready adipogenesis quantification technique

Chengxiang Yuan, Smarajit Chakraborty, Krishna K. Chitta, Subha Subramanian, Tau En Lim, Weiping Han, Bhanu Prakash K.N., Shigeki Sugii

**Additional Methods**

**RNA isolation and RT–qPCR (reverse transcriptase–quantitative polymerase chain reaction)**

RNA was isolated from 3T3-L1 cells during differentiation using TRIZOL protocol (Invitrogen) and cDNA was synthesized using a RevertAid H Minus First strand cDNA synthesis kit (Fermentas) according to manufacturer’s protocol. Quantitative analysis of the gene expression by real-time PCR was carried out using SYBR Green PCR Master Mix on a StepOnePlus Real-Time PCR System (Applied Biosystems). Each real-time PCR (10 µL) contained 4 µL of cDNA, 5 µL of SYBR Green (Applied Biosystems™ Power SYBR™ Green PCR Master Mix (Catalogue number: 4367659).) and forward and reverse primer at a final concentration of 2.5µM. Primer sequences used in the current study are listed in Supplementary Table S3. Transcript levels were normalized to GAPDH transcript levels. Relative fold change in expression was calculated using the ∆∆CT method.

**Analytical method modification to measure lipid droplet sizes**

The fluorescent images of Nile Red staining were processed using MATLAB for background subtraction by estimating background using morphological opening (structural element with disk size set at 20). Estimation of circles form (pixel radius 1 to 20 range, later converted into actual scale in μm) was performed with stringent sensitivity on the background subtracted images using the function of circular Hough transform. Each radius of detected circles was combined to create histograms of lipid droplet radii ranges for all images from each time point.

**Additional Figure Legends**

**Figure S1**

Correlation of adipogenic scores and adipogenic genes expression in 3T3-L1 cells. Gene expression profiles of *PPARγ* (A), *FABP4* (B), *CD36* (C), *ADIPONECTIN* (D) and *LEPTIN* (E) were examined by quantitative RT-PCR. Relative mRNA expression measured was from Day-0 (after over-confluency); -2, -4, -7 and -10 during 3T3-L1 differentiation. The values for the indicated samples are normalized to those at day 0. All the values are averages of data performed in triplicate. Results are presented as means ± SEM; *p < 0.05; **p < 0.01; ***p < 0.001. Correlation between gene expression and Adipo Score is represented with correlation coefficients indicated at the bottom of each corresponding mRNA gene expression.

**Figure S2**

Sizes per lipid droplet from Nile Red staining were estimated with the modified method using background subtraction and circular Hough transform during adipogenesis of 3T3-L1 cells. (A) The representative images processed for analysis of individual lipid droplets (recognized in green) are shown for each time point. Scale bars: 100μm (B) Histograms indicate distribution of individual lipid droplet sizes taken from Nile Red staining images (n=11 each time point). Mean radii with standard deviations (in μm) are indicated in the top right corner for each time point.

**Figure S3**

Adipogenesis time course of human iPSC-derived MSCs. (A) Representative histograms of FATS analysis from one image are shown with adipogenic scores. (B) Adipogenic scores were calculated for each time point (from n=6 wells). Results are presented as means ± SEM.

**Figure S4**

FATS analysis of browning in subcutaneous fat-derived ASCs. (A) The FATS analysis of representative staining images of subcutaneous fat-derived ASCs undergoing normal adipogenesis (left) and browning differentiation (right). Individual nuclei are recognized in squares and highlighted either in red for those with surrounding lipid droplets or in green without those. Scale bars: 100μm (B) Average fluorescence measurements of Nile Red staining were made by spectroscopy from normal and browning adipocytes above (n=5-8). (C) Average adipogenic scores of the same samples by FATS (n=5-8). Results are presented as mean ± standard error of mean (SEM).

**Figure S5**

High-throughput adipogenic screening assay of nuclear receptor ligands using human iPSC-derived MSCs was analyzed by FATS. The new screening result of adipogenesis percentage for the top 4 ligands (A) and the bottom 4 ligands (B) that were identified by 3T3-L1 screening (described in Figure 7 and Table S1) is shown. Histograms indicate representatives from a single well.

**Figure S1**


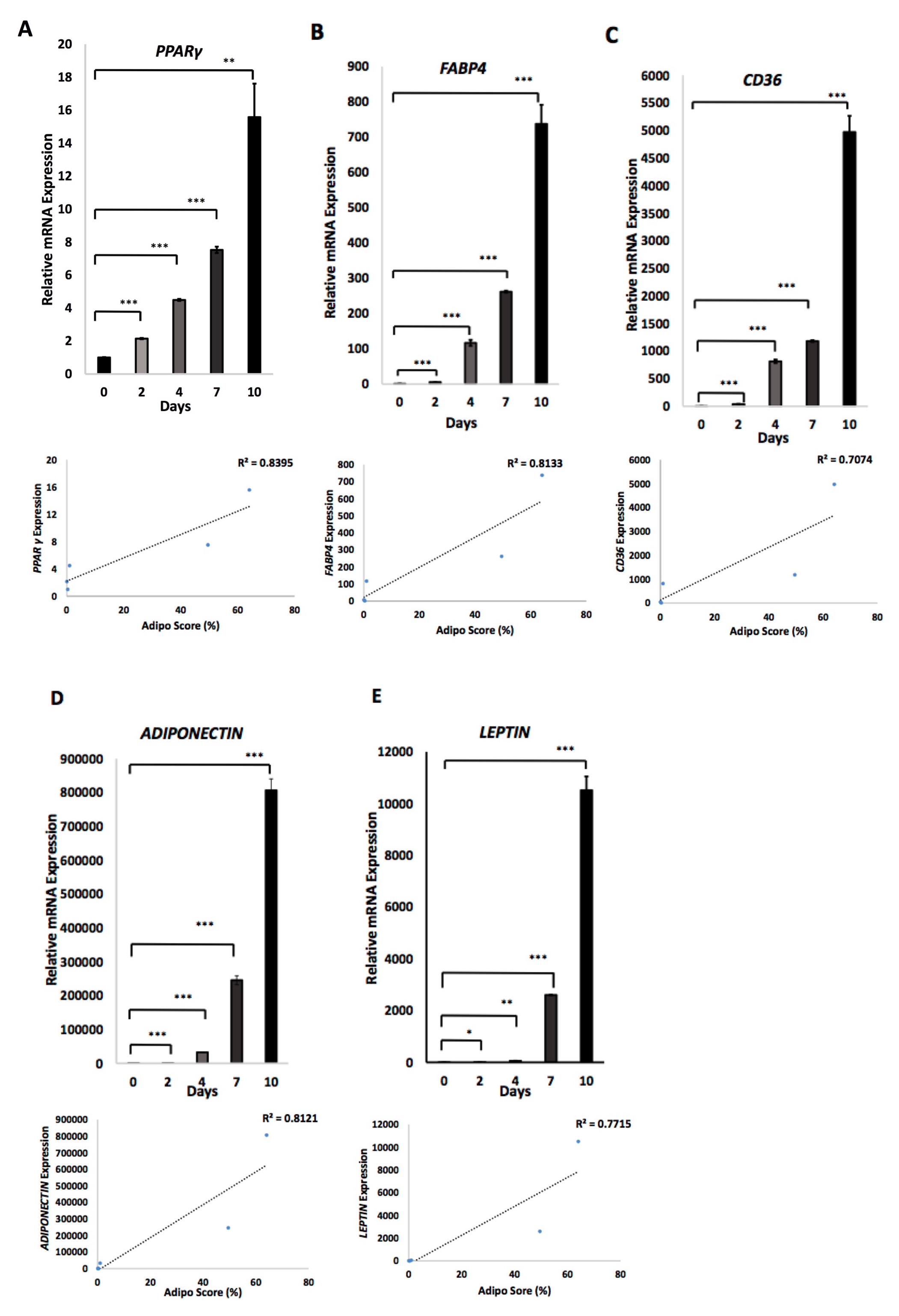


**Figure S2**

**
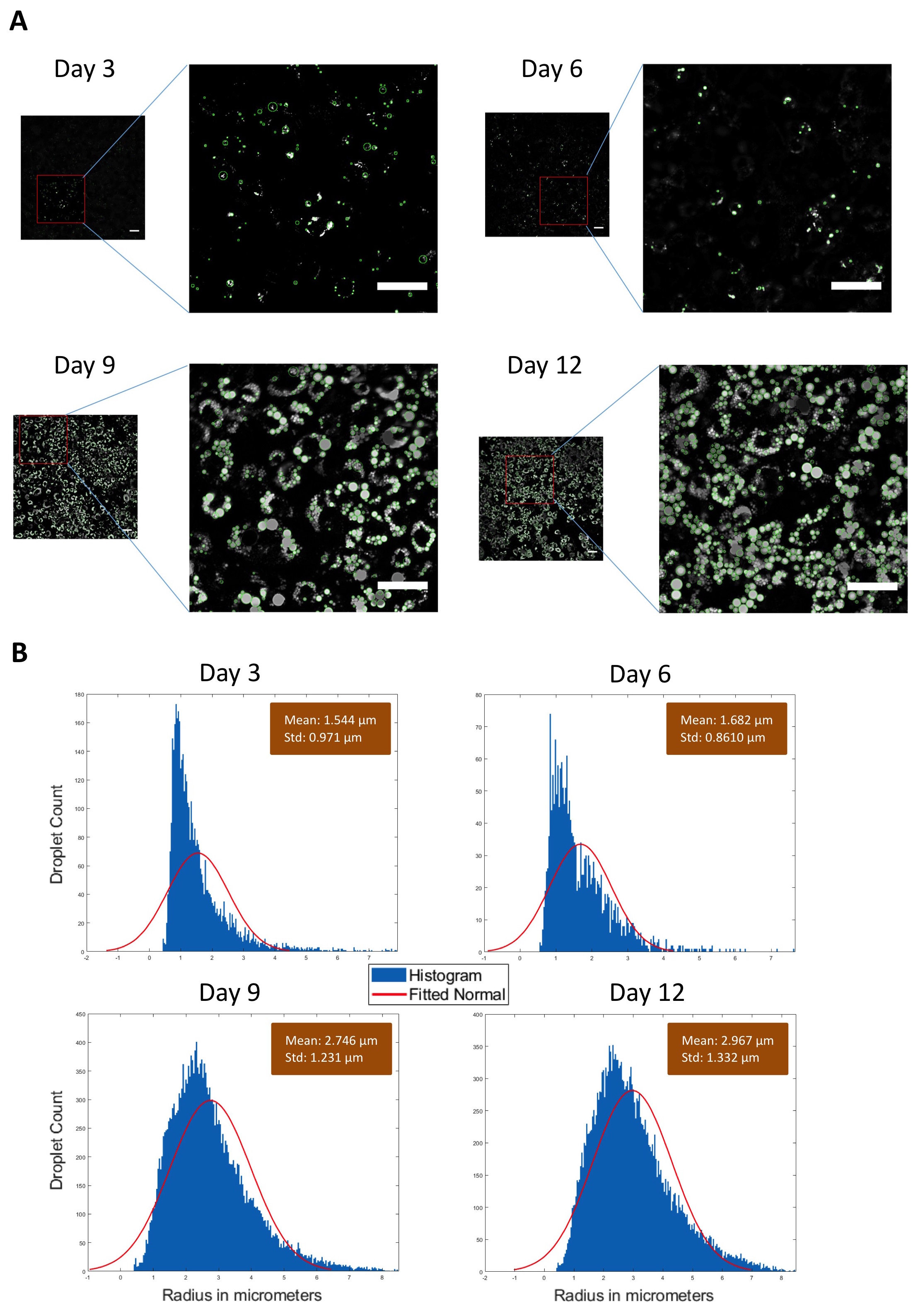
**

**Figure S3**

**
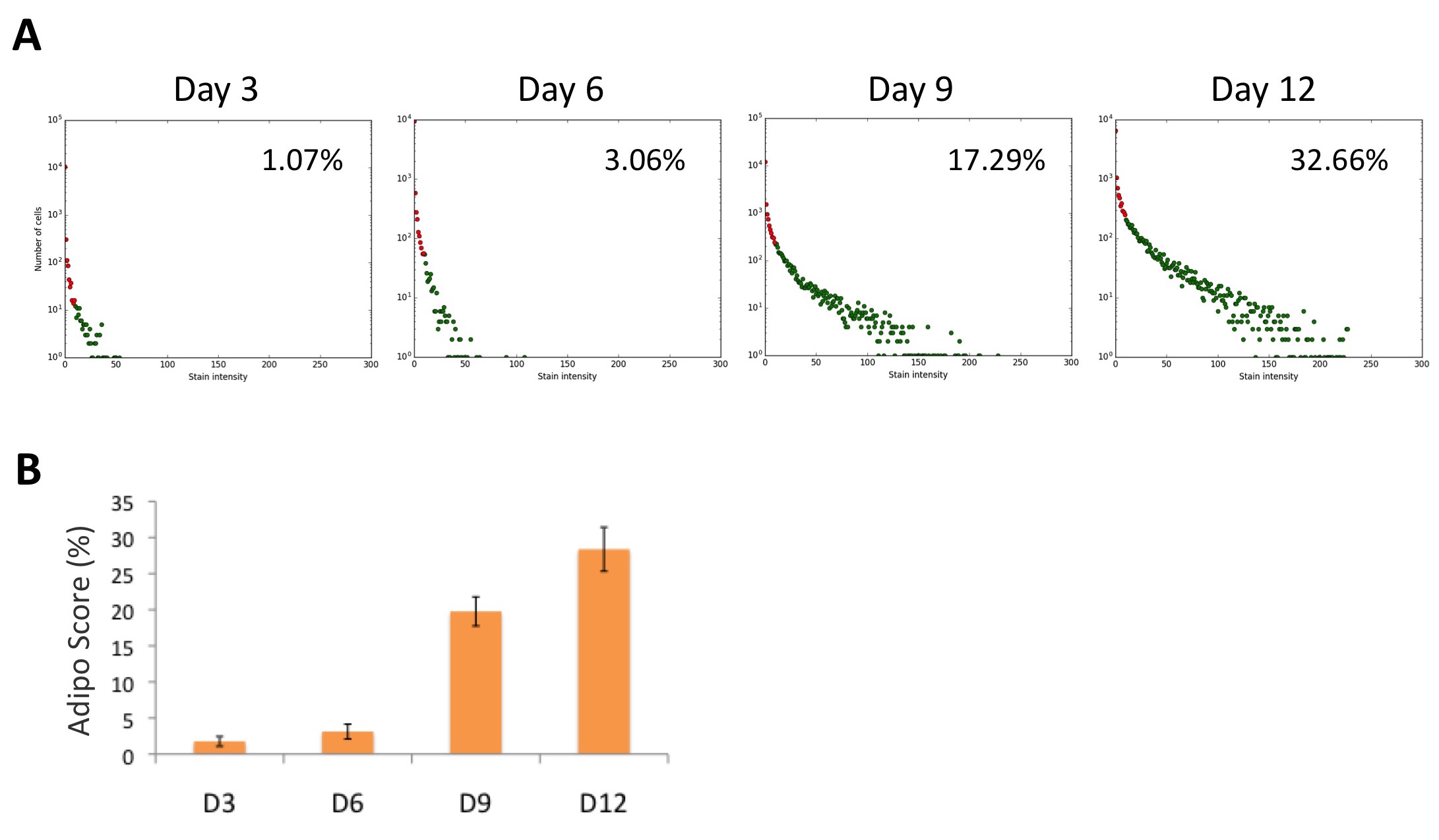
Figure S4**


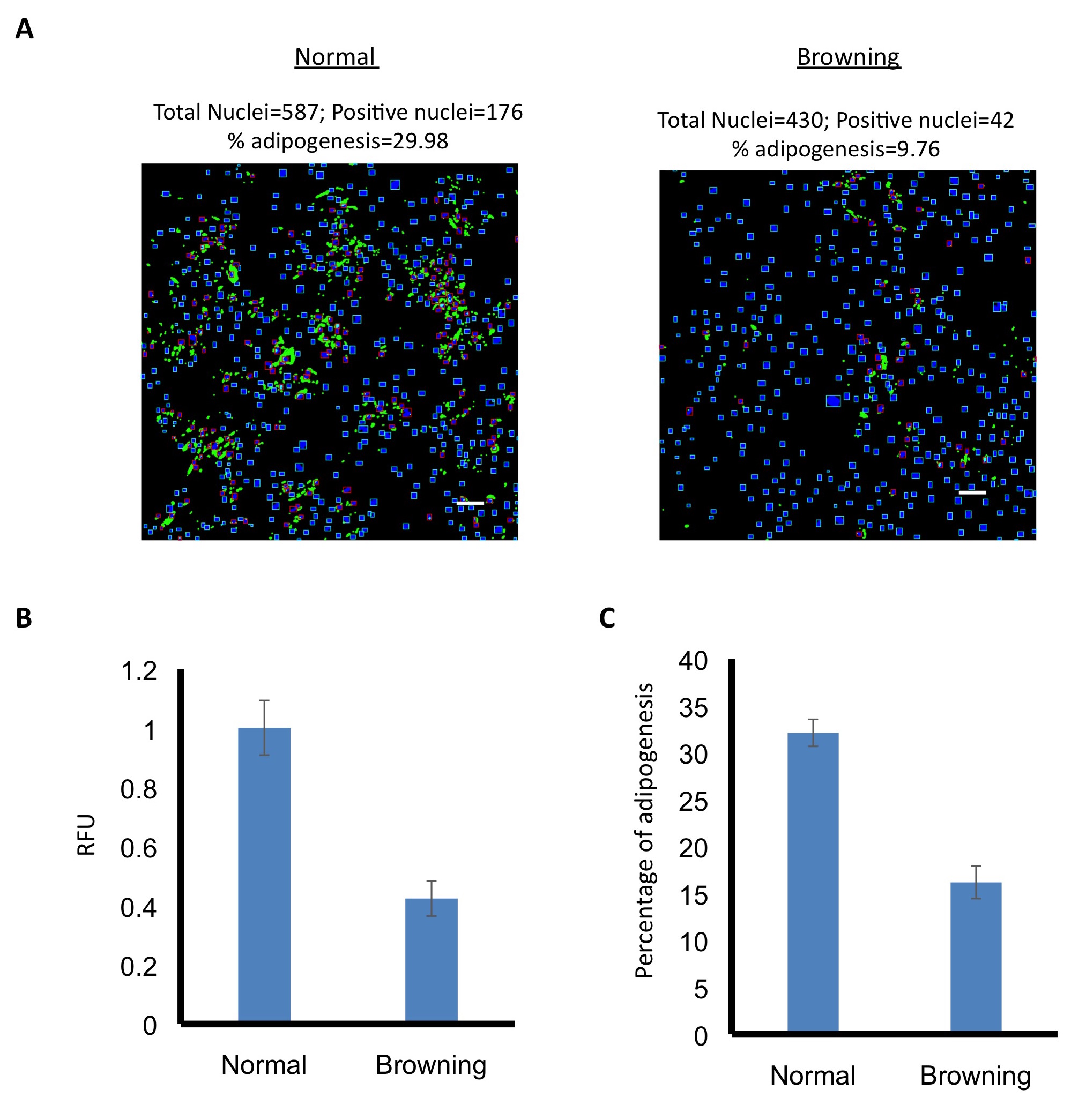


**Figure S5**


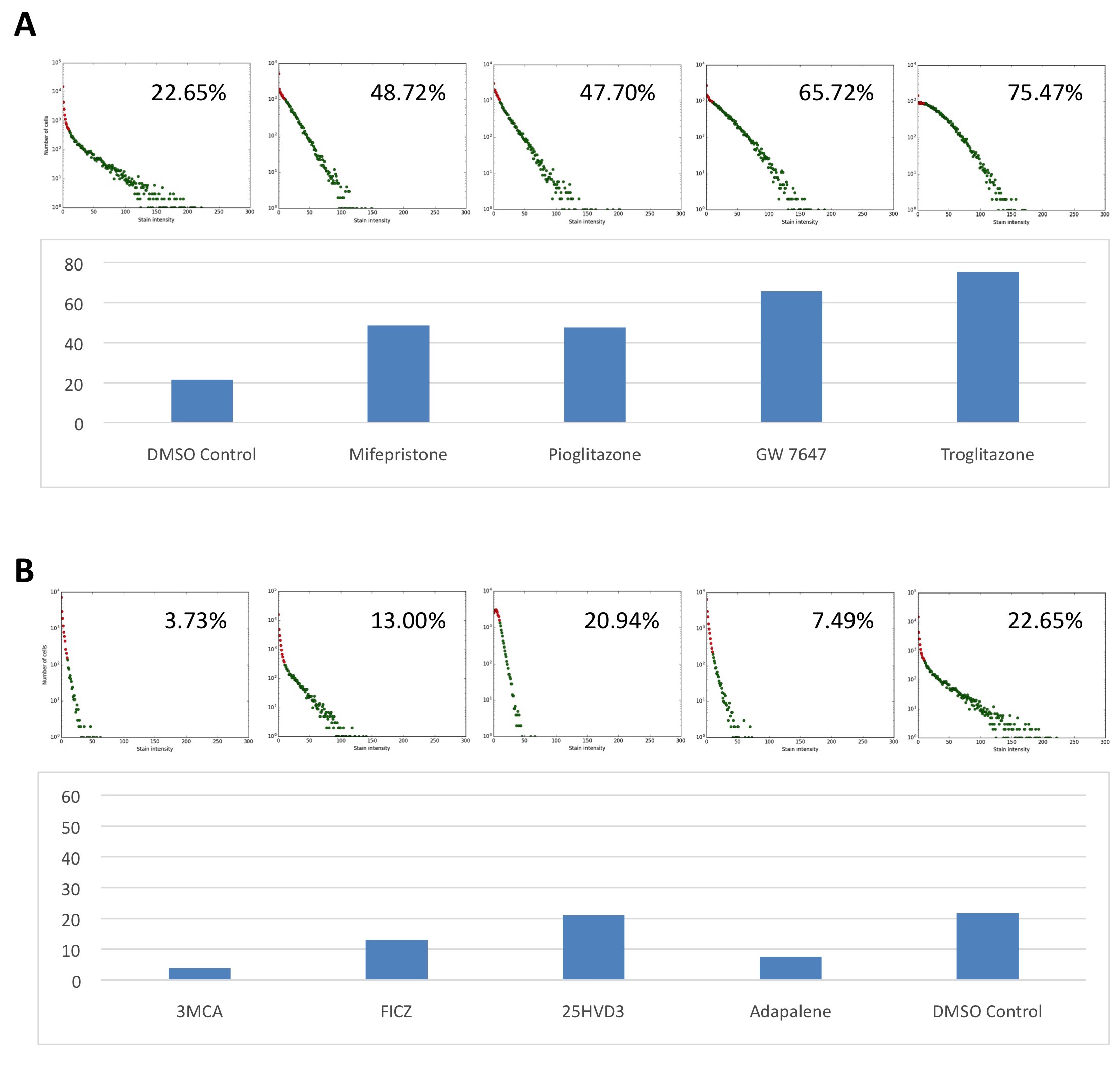
**Table S1**

List of all the nuclear receptor ligands that were tested for high-throughput screening assay for adipogenesis in 3T3-L1 cells. Their activities against target nuclear receptors, and individual and mean adipogenic (AG) scores are shown. Those highlighted in yellow are top and bottom 4 hits, which are represented in the graphs in Figure 7. DMSO control, which was used to normalize AG scores, is shown in red.


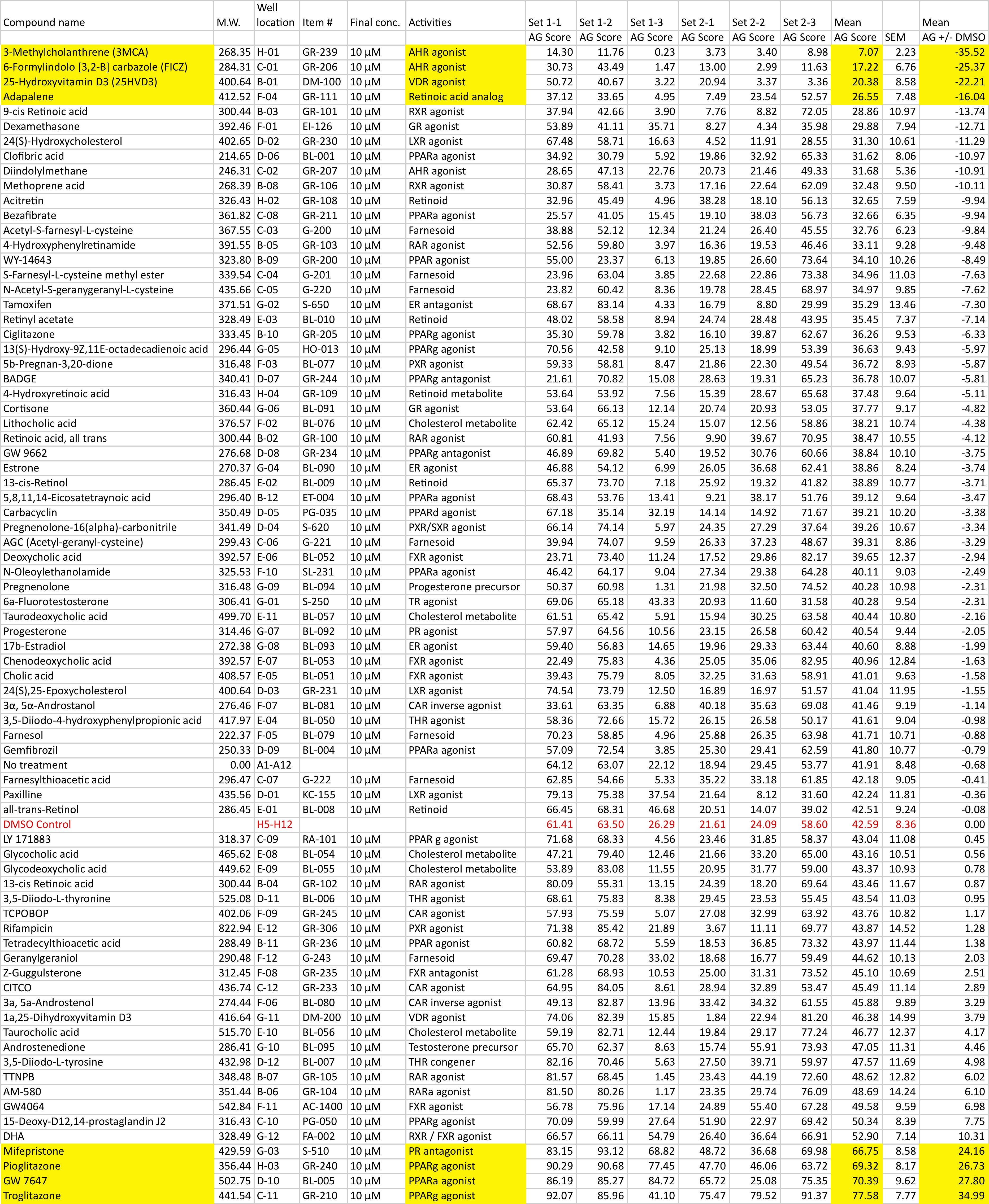


**Table S2**

Results of high-throughput screening assay for adipogenesis of human iPS-derived MSCs in a single plate using nuclear receptor ligands library. Those highlighted in yellow are top and bottom 4 hits from Table S1, which are represented in the graphs in Figure S5.


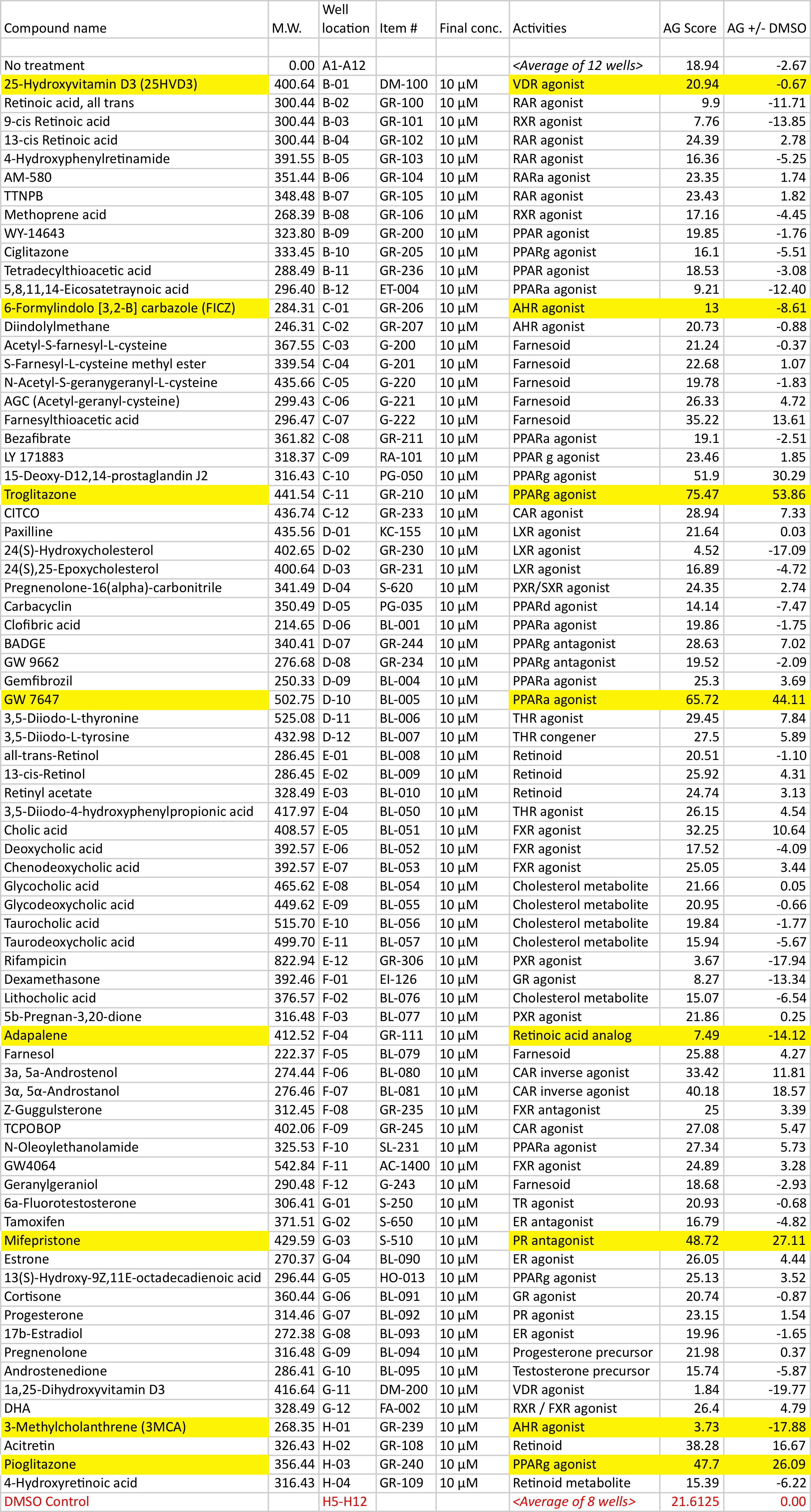


**Table S3**

List of RT-qPCR primers-Oligos 5’ to 3’.

| **Gene** | **Forward** | **Reverse** |
| --- | --- | --- |
| *mGAPDH* | CAAGGTCATCCATGACAACTTTG | GGCCATCCACAGTCTTCTGG |
| *mPPARγ* | GCCCTTTGGTGACTTTATGGA | GCAGCAGGTTGTCTTGGATG |
| *mFABP4* | GATGAAATCACCGCAGACGACA | ATTGTGGTCGACTTTCCATCCC |
| *mCD36* | TAAGCGAGCTAAAGGGAGCA | GTGGTTGAAGGCGTAATGGT |
| *mADIPONECTIN* | GTCAGTGGATCTGACGACACCAA | ATGCCTGCCATCCAACCTG |
| *mLeptin* | CAAGCAGTGCCTATCCAGA | AAGCCCAGGAATGAAGTCCA |
